# The Inclusion of *Alhagi maurorum* in Growing Camel Diet: Effect on Performance, Liver-Related Blood Metabolites, and Antioxidant Status

**DOI:** 10.3389/fvets.2022.863121

**Published:** 2022-03-31

**Authors:** Navid Ghavipanje, Einar Vargas-Bello-Pérez, Mojtaba Afshin, Seyyed Ahmad Hosseini, Alireza Aghashahi, Amir Mansour Vatankhah

**Affiliations:** ^1^Department of Animal Science, Faculty of Agriculture, University of Birjand, Birjand, Iran; ^2^Department of Veterinary and Animal Sciences, Faculty of Health and Medical Sciences, University of Copenhagen, Frederiksberg, Denmark; ^3^Camel and Range Species Research Station of South Khorasan, Birjand, Iran; ^4^Department of Animal and Poultry Nutrition, Animal Science Research Institute, Agricultural Education and Extension Research Organization, Karaj, Iran; ^5^Drug Applied Research Center, Tabriz University of Medical Sciences, Tabriz, Iran

**Keywords:** *Alhagi maurorum*, antioxidants, blood metabolites, growth performance, Sindhi camel

## Abstract

This study determined the effect of dietary inclusion of camelthron [*Alhagi maurorum* (AM)] on the performance, blood metabolites, and antioxidant status of growing camels. A total of 18 Sindhi camel calves of 9–10 months of age and 115 ± 7 kg body weight (BW) were randomly assigned to three diets (with a forage:concentrate ratio of 50:50) that were formulated by partial and total substitution of alfalfa hay with AM as follows: (1) diet without AM (control), (2) diet containing 25% of AM (AM-25), and (3) diet containing 50% of AM (AM-50) (dry matter basis) for 150 days. Dry matter intake (DMI) was recorded daily. The camels were weighed individually on days 0, 30, 60, 90, 120, and 150. Blood samples were collected on days 0, 75, and 150. DMI was increased (*p* = 0.004) with AM-50 feeding followed by AM-25. Total weight gain (*p* = 0.048) and average daily gain (ADG) (*p* = 0.043) decreased with AM-50; however, no differences were observed between the AM-25 and CON groups. Feed cost per kg BW gain tended to decrease (*p* = 0.092) and return per kg BW gain tended to increase (*p* = 0.087) by AM feeding. The plasma triglycerides (TGs) (*p* = 0.046) and cholesterol (CHOL) (*p* = 0.025) concentration were reduced with AM inclusion. Additionally, the AM50-fed camels showed the lowest concentration of aspartate aminotransferase (AST) (*p* = 0.008) and alanine aminotransferase (ALT) (*p* = 0.0036), followed by AM-25. The plasma malondialdehyde (MDA) was depressed (*p* = 0.037) and total antioxidant capacity (TAC) was enhanced (*p* = 0.016) with both the AM-25 and AM-50. Moreover, feeding the AM containing diets led to higher (*p* = 0.004) glutathione peroxidase (GPx) along with a tendency for superoxide dismutase (SOD) (*p* = 0.075) and catalase (CAT) (*p* = 0.094). Overall, feeding camels with AM for up to 25% of their dry matter (DM) diet positively influenced the antioxidant status without severe deleterious effects on performance.

## Introduction

By 2050, the global demand for animal products is projected to increase by 60 to 70% (1). Ongoing food-feed competition, land degradation, and climate change will further generate sustainability challenges to the livestock industry, especially in developing countries, which already face food security challenges (2). In this context, a decision to rear well-adapted livestock species as well as use unconventional plants in the pastures and agrolands could be effective in meeting present and future demands for animal products in a sustainable manner (1, 2).

The dromedary camel (*Camelus dromedarius* or one-humped camel) is one of the most important natural resources in many parts of the world, especially in arid- and semi-arid areas (3, 4); it plays a great economic and social role in these regions, where the common livestock species cannot be efficiently reared (5). Hence, camels are seen as an opportunity for sustainable livestock production (3, 4). The worldwide camel population is about 35.5 million heads, producing about 0.557 million tons of meat per year (6). Most camels in the world are usually reared on natural grazing conditions; however, the camel farming system is changing from the traditional extensive to the modern intensive systems (4–9). However, there is a lack of scientific information on the nutritional management and performance of camels as well as evaluation of dominant pasture plants grazed by camels. Moreover, the trend is to increase the use of natural feedstuffs due to consumer concerns over the safety of animal products (10). The pasture and range plants are naturally rich in bioactive molecules, which could be used as a feeding strategy to improve ruminant products quality by enhancing the antioxidant capacity and also to reduce the cost of production (10).

*Alhagi maurorum* (AM), also known as camelthorn, is a halophyte plant that belongs to the *Fabaceae* family and has been placed under the *Papilionaceae* subfamily (11, 12). AM is a spiny deep-rooted perennial shrub with roots that can reach six or seven feet into the ground, and it is widely distributed in Central Asia, North America, Europe, Mediterranean, East Asia, North Africa, South Africa, and Northwestern China (12, 13). This plant normally grows in dry lands associated with low rainfall and in areas with high salinity and alkalinity (12–14). AM has been known as a potential medicinal plant that is traditionally used for treating numerous diseases such as gastroenteritis, diarrhea, inflammations, and liver disorders (12, 15). Currently, pharmacological studies have confirmed that AM is rich in phenolic, alkaloid, and flavonoid compounds, which are accompanied by antioxidant, antitumor, and antineuroinflammatory effects, hepatoprotective effects, renoprotective effects, and immune regulation (12–15). It is well-established that AM extracts enhance the antioxidant status of rats (13) and possess *in-vitro* radical scavenging activity (12). In addition, Alghasemi et al. (16) have shown the hepatoprotective effects of AM plants in experimentally liver injury-induced rats, which were associated with lower plasma alanine aminotransferase (ALT), aspartate aminotransferase (AST), and bilirubin. Although the potential of AM as a low-cost feed for replacing conventional forages in ruminant diets has been suggested (17), its nutritional value has received little attention. In previous reports (18, 19), crude protein (CP), neutral detergent fiber (NDF), acid detergent fiber (ADF), and nonfiber carbohydrate (NFC) contents of AM were reported to be 11.5, 49.5, 32.9, and 30.2% [dry matter (DM) basis], respectively. The results of gas chromatography-mass spectrometry (GC-MS) analysis (20) also revealed that AM consisted of a complex mixture of different substances, with ketones (5.2%), acid derivatives (1.8%), terpenoids (26.8%), hydrocarbons (19.3%), heterocyclics (5.2%), and aldehydes (0.2%). Saleem et al. (21) have identified the total phenolic [105.91 mg gallic acid equivalents (GAE)/g extract] and flavonoid [2.27 mg rutin equivalents (RE)/g extract] contents of AM methanolic extracts using ultra-high-performance liquid chromatography-MS (UHPLC-MS) analysis, which can be correlated to its more substantial antioxidant potential. Kazemi and Bezdi (18) have been shown that AM could be used up to 250 g/d in lactation ewe's diet. Also, a study reported that the AM is one of the most palatable plants for camels (19).

Despite the promising prospects of camel rearing and its effects on boosting food security, very little attention has been paid to its nutritional practices. In intensive camel production, reducing feeding costs by replacing conventional forages (i.e., alfalfa hay) with AM might guarantee the profitability of the system, due to its low cost (18, 19) and availability (17). However, until now, the effect of AM inclusion in the growing camel diet is unknown. Thus, the objective of this study was to investigate the possibility of replacing alfalfa hay with AM in diets fed to Sindhi growing camels and to determine the effects on growth performance, blood metabolites, and antioxidant status. We hypothesized that AM inclusion in growing camel diets will promote a favorable antioxidant profile due to its bioactive compounds along with reduced production costs without deleterious effects on performance.

## Materials and Methods

### Design, Animals, and Diets

This experimental study was performed following the guidelines of the Iranian Council of Animal Care (22) (protocol ID 19293). This study was carried out at the “Camel and Rangeland Research Station of South Khorasan,” located at 37.42° N and 57.31° E longitude, 1,491 m above sea level, in Birjand, Iran.

In total, 18 male 9–10-month-old Sindhi camel calves with a mean body weight (BW) of 115 ± 7 kg were randomly assigned to the three treatment diets in a completely randomized design with six animals per treatment, each kept in an individual shaded pen (3 m × 3 m) for 150 days preceded by an adaptation period of 14 days. During the adaptation period, all the camels were treated for external and internal parasites, vaccinated against enterotoxemia (Enteroprotect P 100, Razi Vaccine and Serum Research Institute, Iran), and fed the same diet (control diet). The diets were formulated by partial and total substitution of alfalfa hay with AM and included the following: (1) control, with no AM (CON), (2) AM-25, containing 25% AM, and (3) AM-50, containing 50% AM (DM basis) (Table 1). The whole AM plant was hand harvested from the Heydarabad (Nehbandan, South Khorasan, Iran) rangelands in June (at flowering growth stage) of 2021 and then dried and stored in a dark place until used. Diets consisting of 50 forage and 50% concentrate were formulated to meet energy and protein requirements of growing camels (23, 24), which were isocaloric (contain 2.47 Mcal/kg ME) and isonitrogenous (contain 114 g/kg CP). Throughout the experiment, the total mixed ration (TMR) diets were offered *ad libitum*, with feeding levels designed to ensure a daily refusal margin of 10% with free access to the water. During the experiment, camels were fed individually twice daily (0800 and 1700 h), and amounts of diet fed and refusals were weighed daily to determine DM intake (DMI) by subtracting the quantity fed minus refusals multiplied by the DM content of the TMR.

**Table 1:** Ingredients and nutrient composition [dry matter (DM) basis] of experimental diets.

	**Diets*^a^***		
	**CON**	**AM-25**	**AM-50**
**Ingredient (% of DM)**			
Alfalfa hay	45	25	0
Alhagi maurorum^*b*^	0	25	50
Wheat straw	5	0	0
Ground barley grain,	22	22	22
Ground corn grain,	15	13	11
Soybean meal	2	4	6
Canola meal	3	3	3
Cotton seed meal	1	1	1
Wheat bran	5	5	5
Sodium bicarbonate	1	1	1
Salt	0.5	0.5	0.5
Minerals and vitamins premix^*c*^	0.5	0.5	0.5
Chemical composition			
ME, Mcal/kg of DM	2.47	2.47	2.46
Crude protein (% DM)	14.1	14.1	14.1
Ether extract (% DM)	2.50	2.70	2.80
Neutral detergent fiber (% DM)	33.1	33.3	33.6
Acid detergent fiber (% DM)	21.5	21.8	22.0
Non-fibrous carbohydrates (% DM)^*d*^	44.9	44.8	44.5

a *CON, AM-25 and AM-50 contained 0, 25, and 50% AM (DM basis), respectively*.

b *Alhagi maurorum (AM) contain of 93.78 DM, 9.55 CP, 2.37% ether extract, 10.2 ash, 46.2 NDF, 37.2% ADF, 10.6 total phenols, 7.42 total tannins, 2.07 condensed tannins, and 5.41% hydrolysable tannins (DM basis)*.

c *Containing vitamin A (250,000 IU/kg), vitamin D (50,000 IU/kg), vitamin E (1,500 IU/kg), manganese (2.25 g/kg), calcium (120 g/kg), zinc (7.7 g/kg), phosphorus (20 g/kg), magnesium (20.5 g/kg), sodium (186 g/kg), iron (1.25 g/kg), sulfur (3 g/kg), copper (1.25 g/kg), cobalt (14 mg/kg), iodine (56 mg/kg) and selenium (10 mg/kg)*.

d *Non-fibrous carbohydrates (NFC) were estimated according to the equation: NFC = 100 – (NDF + CP + EE + Ash)*.

### Sample Collection and Measurements

The TMR and refusal samples were taken during the trial (on days 0, 30, 60, 90, 120, and 150) before the morning feeding and frozen at −20°C for later analysis. The camels were weighed individually before the morning feeding on days 0, 30, 60, 90, 120, and 150 to determine the average daily gain (ADG). Feed conversion ratio (FCR) and feed efficiency (FI) was calculated as follows: FCR = [DMI, (kg/d)/ADG, (kg/d)]; FCR = [ADG, (kg/d)/DMI, (kg/d)]. An estimate was also made of the feeding cost per kg of body weight gain for the experimental groups. Blood samples were collected after overnight fasting through the jugular vein using tubes containing heparin lithium as an anticoagulant (Avapezeshk, Tehran, Iran) on days 0, 75, and 150. Plasma samples were obtained by centrifuging the blood tubes for 10 min at 3,000 × g and then were frozen at −20°C until analysis.

### Laboratory Analysis

Samples of TMR and orts were separately pooled and grounded in a hammer mill with a 1-mm screen (Arthur Hill Thomas Corporation, Philadelphia, Pennsylvania, USA) and analyzed (three replicates) for DM (930.15), CP (Kjeldahl N × 6.25, 990.03), ether extract (EE) (945.16), and ash (967.05) according to Association of official analytical collaboration (AOAC) (25). The NDF and ADF content of samples were analyzed (Fibertec 1010, Tecator, Sweden) according to Van Soest et al. (26).

The plasma samples were analyzed for glucose (Glu) (mg/dl), total protein (TP) (g/dl), creatinine (Cr) (mg/dl), albumin (Alb) (g/dl), triacylglycerol (TG) (mg/dl), cholesterol (CHOL) (mg/dl), high-density lipoprotein (HDL) (mg/dl), blood urea nitrogen (BUN) (mg/dl), ALT (U/L), AST (U/L), and alkaline phosphatase (ALP) (U/L) using commercial kits (Pars Azmun Corporation Ltd., Tehran, Iran) by an autoanalyzer (BT 1,500, Biotecnica Instruments SpA, Rome, Italy) according to the manufacturer's instructions. Plasma total antioxidant capacity (TAC) (mmol/l) was measured using 2,2'-azino-bis (3-ethylbenzothiazoline-6-sulfonic acid) (ABTS) radical formation with the commercially available Randox Kit (Randox Laboratories Ltd., Crumlin, County Antrim, UK). Plasma malondialdehyde (MDA) (nmol/ml) level was determined using the thiobarbituric acid reactive substance (TBARS) according to a study by Placer et al. (27). The enzymatic activities of glutathione peroxidase (GPx) (U/ml), superoxide dismutase (SOD) (U/ml), and catalase (CAT) (U/ml) were measured using the Randox Kit (Randox Laboratories Ltd., Crumlin, County Antrim, UK) according to the manufacturer's instructions.

### Statistical Analysis

A completely randomized design with three treatments (diets) and six replicates (camels) was used for this study. All the data were statistically analyzed using the MIXED procedure of SAS (version 9.2, SAS Institute Incorporation, Cary, North Carolina, USA) (28) for repeated measures. The fixed effects in the model were: the dietary treatment (diet), the time of storage (time), and their interaction (diet × time), while camel was included as a random factor. Initial body weight and blood parameters of camels were used as covariates in the model. Least-square means (LSM) were calculated and tested for differences by Tukey's test. A polynomial contrasts analysis was employed to determine the linear and quadratic effects of AM levels. Differences in LSM were significant at *P* ≤ 0.05 and *P* ≤ 0.10 was considered as a tendency.

## Results

### Growth Performance and Feeding Cost

The animal performance results are given in Table 2. DMI was increased with the inclusion of 25 and 50% (DM basis) AM to diet. The average initial BW (IBW) of the camels from each treatment had no difference (*p* = 0.652). The final BW (FBW) of animals fed AM diets tended to decrease (*p* = 0.096). The camels fed AM-25 and AM-50 had approximately 1.01 and 4.4% lower FBW compared to the CON group. Likewise, total weight gain (*p* = 0.048) and ADG (*p* = 0.043) decreased with AM-50; however, no differences were observed between the AM-25 and CON groups. In addition, higher FCR values were found in AM-50 treatment (*p* = 0.018). Feed cost per kg BW gain tended to decrease (*p* = 0.092) by the increasing inclusion levels of AM in the experimental diets. A tendency to increase (*p* = 0.087) in return per kg BW gain was also observed with AM-25 and AM-50 feeding.

**Table 2:** DM intake, growth performance, and feeding cost of growing camels fed experimental diets^*A*^.

**Parameters^B^**	**Diets^C^**			**SEM^E^**	***p*** **-values**		
	**CON**	**AM-25**	**AM-50**		**Diet**	**Linear**	**Quadratic**
DMI (kg/d)	4.45^b^	4.50^ab^	4.58^a^	0.029	0.004	0.047	0.548
IBW	117.2	113.9	117.1	3.31	0.652	0.825	0.827
FBW (kg)	185.3	183.4	177.2	3.48	0.096	0.582	0.455
Total BW gain (kg)	68.68^a^	67.50^a^	60.28^b^	2.43	0.048	0.286	0.305
ADG (g/d)	0.485^a^	0.450^a^	0.402^b^	0.011	0.043	0.245	0.307
FCR	9.78^b^	10.03^b^	11.53^a^	0.318	0.018	0.111	0.213
FE	0.102^a^	0.099^ab^	0.087^b^	0.003	0.029	0.404	0.466
Feed cost per kg BW gain (US$)^D^, ^E^	1.72	1.59	1.60	0.041	0.092	0.243	0.308
Return per kg BW gain (US$)^D^, ^E^	0.683	0.796	0.751	0.060	0.087	0.260	0.352

*Within row, different letters (a, b) indicate difference between diets (p ≤ 0.05)*.

A *Values are least-square means*.

B *DMI, dry matter intake; IBW, initial body weight; FBW, final body weight; ADG, average daily gain; FCR, feed conversion ratio; FE, feed efficiency*.

C *CON, AM-25 and AM-50 contained 0, 25, and 50% Alhagi maurorum (AM) (DM basis), respectively*.

D *Each kilogram of alfalfa and AM was 0.21 and 0.14 USD, respectively*.

D *Calculations are made with the following exchange: 1 USD = 271,358 IR Rials*.

E *SEM, pooled standard error of the mean*.

### Blood Metabolites

No significant effects between treatments were observed regarding Glu, TP, Cr, hemoglobin (Hb), and BUN (Table 3). The TG (*p* = 0.046) and CHOL (*p* = 0.025) concentrations were lower for AM-25 and AM-50 compared with CON. The Alb levels were increased (*p* = 0.047) with the increasing AM inclusion in the growing camel's diet. Additionally, the AM50-fed camels showed the lowest concentration of AST (*p* = 0.008) and ALT (*p* = 0.0036), followed by AM-25. Likewise, a tendency (*p* = 0.008) to decrease was also observed for ALP with increasing AM inclusion to the diets.

**Table 3:** Liver-related blood parameters of growing camels fed experimental diets^*A*^.

**Parameters^B^**	**Diets^C^**			**SEM^D^**	***p*** **-values**		
	**CON**	**AM-25**	**AM-50**		**Diet**	**Linear**	**Quadratic**
Glu (mg/dL)	95.0	99.3	104	3.58	0.281	0.444	0.670
TG (mg/dL)	17.3^a^	13.0^b^	11.7^b^	1.27	0.046	0.033	0.324
CHOL (mg/dL)	18.3^a^	15.3^b^	14.7^b^	0.745	0.025	0.070	0.248
TP (g/dL)	5.13	5.20	5.00	0.191	0.676	0.668	0.498
Cr (mg/dL)	1.04	0.847	0.826	0.104	0.335	0.324	0.514
Alb (mg/dL)	3.10^b^	3.27^ab^	3.70^a^	0.135	0.047	0.092	0.445
Hb (g/dL)	8.70	11.5	10.0	1.23	0.348	0.191	0.251
BUN (mg/dL)	24.7	23.0	22.7	4.13	0.757	0.864	0.919
AST (mg/dL)	137^a^	101^b^	85.7^b^	7.80	0.008	0.023	0.201
ALT (mg/dL)	22.0^a^	19.0^ab^	17.3^b^	0.962	0.036	0.085	0.592
ALP (mg/dL)	207	172	152	18.2	0.093	0.344	0.724

*Within row, different letters (a, b) indicate difference between diets (p ≤ 0.05)*.

A *Values are least-square means*.

B *Glu, Glucose; TG, triacylglycerol; CHOL, cholesterol; TP, total protein; Cr, creatinine; Alb, albumin; Hb, hemoglobin; BUN, blood urea-N; AST, aspartate aminotransferase; ALT, alanine aminotransferase; ALP, alkaline phosphatase*.

C *CON, AM-25 and AM-50 contained 0, 25, and 50% Alhagi maurorum (AM) (DM basis), respectively*.

D *SEM, pooled standard error of the mean*.

### Antioxidant Status

The AM-25 and AM-50 treatments showed a 19.4 and 27.6% lower (*p* = 0.037; Table 4) plasma MDA content compared to CON. Moreover, the plasma TAC of growing camels was increased (*p* = 0.016) with increasing the AM inclusion to diets, which led by AM-50 following AM-25. Likewise, the inclusion of 25 and 50% (DM basis) AM to the diets led to higher (*p* = 0.004) GPx concentration. In addition, SOD (*p* = 0.075) and CAT (*p* = 0.094) concentrations were also tended to enhance by AM inclusion to the diets.

**Table 4:** Antioxidant status of growing camels fed experimental diets^*A*^.

**Parameters^B^**	**Diets^C^**			**SEM^D^**	***p*** **-values**		
	**CON**	**AM-25**	**AM-50**		**Diet**	**Linear**	**Quadratic**
MDA (nmol/ml)	1.70^a^	1.37^b^	1.23^b^	0.098	0.037	0.143	0.437
TAC (mmol/L)	0.330^b^	0.380^ab^	0.417^a^	0.014	0.016	0.018	0.723
GPx (U/mL)	93.3^b^	98.8^ab^	105^a^	1.50	0.004	0.024	0.687
SOD (U/mL)	15.6	18.7	21.7	1.47	0.075	0.445	0.779
CAT (U/mL)	12.7	15.3	15.9	1.03	0.094	0.191	0.837

*Within row, different letters (a, b) indicate difference between diets (p ≤ 0.05)*.

A *Values are least-square means*.

B *MDA, Malondialdehyde; TAC, total antioxidant capacity; GPx, glutathione peroxidase; SOD, superoxide dismutase; CAT, catalase*.

C *CON, AM-25 and AM-50 contained 0, 25, and 50% Alhagi maurorum (AM) (DM basis), respectively*.

D *SEM, pooled standard error of the mean*.

## Discussion

Given the challenges facing worldwide animal agriculture, namely, limited availability of natural resources, ongoing climatic changes, and food–feed–fuel competition, particularly in the arid and semi-arid regions, which are subjected to periodical drafts and erratic patterns of rainfall, severely affecting conventional livestock production (i.e., namely cattle and sheep), camels may become an important way to enhance food and feed security (4, 7). Therefore, camels have been subjected to an increasing research interest (3); however, there is still a lack of information on their growth performance following feeding with halophyte plants, which also is a main feature of camels (16, 19). The AM has been shown to have great potential as animal feed (18, 19), but to the best of our knowledge, no investigation has been addressed AM in growing camel. In this study, we found that AM inclusion in a growing camel diet improved antioxidant status and reduced production costs without severe deleterious effects on performance.

### Growth Performance

The DMI in this study is consistent with an earlier report (29) demonstrating a daily 4.36 kg DMI for growing camels aged 8 to 12 months (with an average 120 kg BW) during the 90-day feeding period. Also, it has been reported that camel DMI mainly depends on the type and availability of forages as well as their quality (19, 30). Although most camel studies have not reported DMI based on standard diets, the DMI of growing camels has been reported to be between 1.6 and 3 kg per 100 kg live weight (30, 31). Thus, the observed DMI values were within the normal range for dromedary growing camels. The inclusion of 25 and 50% AM (DM basis) to the diets resulted in 1.2 and 2.9% higher DMI compared to the CON group, respectively. Our results agree with a study by Kazemi and Bezdi (18) and Karamshahi Amjazi et al. (32) who found significantly higher DMI in ewes fed AM diets. In addition, it has been reported that there is a contraction between the type of pasture and feed behavior in dromedary camels (33), where plants with higher palatability have been associated with higher camels intake (19, 33). In line with the present data, a study (19) that aimed to investigate the nutritive value of some herbage for dromedary camels in the central arid zone of Iran reported that the AM classified as the most palatable plants for these animals. Camels have been shown to prefer prickly shrubs such as AM to other species (34). AM appears to enhance DMI, which is mainly associated with the higher palatability of AM for camel as well as camel preference for the consumption of thorny plants. Consistently, it has been well-documented that camels consume more prickly plants, which is related to the inherent adaptation of this animal and its grazing behavior, which make these plants more palatable (34, 35).

It has been shown (31, 36) that the growth rate of the camel calves is higher in the early stages of growth (up to 6 months old) and then decrease with puberty. Growth performance data presented in this study are similar to results reported in the literature using dromedary camels (37). ADG in dromedary camel calves is lower than cows and limited to 500 g/day (30). Wilson (36) reported a 326 g/day weight gain for 1-year-old camels that were fed diets containing 50 forage and 50% concentrate for 175 days, which is almost consistent with the present results.

Our results indicated that the inclusion of AM up to 25% DM did not affect ADG, FCR, and FE. In contrast, camels fed the greatest AM level (up to 50% DM of diet) had a reduction in total BW gain (~12.1% compared to the CON group and 10.6% compared to inclusion with 25% AM), resulting to lower ADG and higher FCR. It is well-accepted (38, 39) that the reduction in diet digestibility negatively affects weight gain. In this study, we did not investigate the digestibility of diets. However, it is known (18, 32, 34) that AM had lower digestibility compared to alfalfa hay. Similarly, Karamshahi Amjazi et al. (32) reported that the lower digestibility of AM compared to alfalfa hay may relate to its higher NDF, ADF, and lignin content. In this regard, Kazemi and Bezdi (18) showed that replacing the dietary forage with AM resulted in decreased nutrient digestion and decreased BW change of ewes. In this study, lower ADG in animal fed AM up to 50% DM of diet is probably attributed to decrease nutrient digestion that further increases FCR. In line with this, Karamshahi Amjazi et al. (32) reported that the apparent digestibility of DM and CP was decreased with AM silage inclusion in a sheep diet. Moreover, total BW gain and ADG in AM-25 were numerically higher and this could provoke different rates of digestion, which might enhance the efficiency of energy utilization and consequently enhance growth rate (40). Analysis of feeding costs showed that inclusion of AM to diet led to a sensible reduction of the feed cost per kg BW gain (7.6 and 6.9% reduction with the inclusion of 25 and 50% AM and DM basis). Altogether, our results indicated a favorable effect of AM inclusion on the DMI of growing camels, which was probably associated with its higher palatability as well as camel preferences for consume AM. The ADG was decreased with AM-50. However, there were no differences between AM-25 and CON in regard of ADG and FCR that along with lower feed cost per kg BW gain make AM-25 rationalization in growing camel diet.

### Blood Metabolites

The current values of blood parameters were in line with previously published data on fattening camels (20). The inclusion of AM in camel diets led to a reduction of TG and CHOL concentrations. In this regard, the inclusion of 1.5, 3.0, and 4.5% (DM basis) AM powder in laying hens diet reduced in plasma TG. Also, Riasi et al. (41) reported that the inclusion of halophyte plants in sheep diets reduced the plasma concentration of TG and CHOL. In support of earlier findings, it has been shown that the ethanolic extracts of AM (at a dose of 200 and 300 mg/kg) significantly reduce the level of blood CHOL and TG in diabetic Wistar rats (42). Some previous studies (15, 43) indicate that the lipid-lowering effects of AM may relate to its large number of active compounds such as alkaloid, peptidoglycan, terpenoid, amino acid, and, especially, flavonoids.

Overall, feeding of both the AM-25 and AM-50 mitigates the concentration of liver enzymes, namely, AST, ALT, and ALP, along with the increase in Alb. The activities of the aforementioned biomarkers were discussed together, as they are commonly used to study liver function (44). The reduction in AST, ALT, and ALP of the AM-containing groups implies the enhanced liver function in these camels. Consistent with our results, Kuerbanjiang et al. (45) showed that the AM significantly alleviated alcohol-induced liver injury in mice by reducing serum ALT and AST. Moreover, Alqasoumi et al. (15) have shown that the ethanolic extract of aerial parts of AM at a dose of 500 mg/kg decreased liver enzymes and increased plasma Alb in CCl_4_-induced liver injury in rats. In agreement with our results, it has been shown (5, 46) that using AM extract reduced the liver enzymes (AST and ALT) in mice with liver injury, which is associated with high levels of flavonoids and bioactive polysaccharides in this plant. The AM is known to reduce the gene expression of Toll-like receptor 4 (TLR4), inhibiting the release of tumor necrosis factor-α (TNF-α) as well as preventing further signal transmission and reducing the efficiency of endotoxin signal transduction, which led to hepatoprotective effects (5). In addition, previous studies (5, 15) suggested that the hepatoprotective mechanism of AM has been partly attributed to the reduction of oxidative stress and inhibition of the expression of cytochrome P450 2E1 (CYP2E1). Our finding suggests that AM not only favors a plasma lipid profile, but it could also improve liver activity of growing camels.

### Antioxidant Status

The dynamic balance between oxidation and antioxidant activity in the circulatory system plays a critical role in the health and productive performance of livestock (47). The rich functional compounds in nature-derived feedstuffs make them an attractive component to improve the antioxidant status of animals, thereby improving the quality of products (47–49). Currently, consumers are interested in safe and natural foods of animal origin and in some cases, they are also willing to pay a premium price for them (49). TAC reflects the cumulative effects of enzymatic and non-enzymatic antioxidants present in plasma and body fluids (47, 48), whereas MDA is generated because of lipid peroxidation that could be measured as a biomarker of oxidative stress (47). Mammalian enzymatic antioxidation against free radical damage is mainly facilitated by the activities of SOD, GPx, and CAT (47). The results obtained regarding the antioxidant status in this study are consistent with previously published data on growing camels (23). Our results demonstrated that AM had positive effects on antioxidant status, as indicated by enhanced blood TAC, GPx, SOD, and CAT activity and depressed MDA levels. In confirmation, the antioxidant activity of AM has been proved by *in-vitro* and *in-vivo* studies (13, 15). In this regard, Peluso et al. (50) reported that the treatment of diabetic rats with AM extracts improved the plasma antioxidant status by increasing the activities of SOD, GPx, and glutathione transferase (GST). Likewise, AM extract (100 mg/kg body weight) decreased plasma MDA levels in rats (51). AM is rich in biologically active phytochemicals such as phenolics, flavonoids, alkaloids, and polysaccharides along with different essential minerals, proteins, and lipids, which has turned it into a powerful antioxidant (13, 15). Natural plant feeds containing phenolic, flavonoid, and alkaloid substances can prevent peroxidation by scavenging free radicals or by activation of antioxidant enzymes such as glutathione reductase, GPx, and SOD (49). A study (52) reported that the aqueous extract of AM was appraised by reducing MDA levels using thiobarbituric acid assay. Laghari et al. (12) demonstrated high antioxidant activity of AM plant using 2,2-diphenyl-1-picrylhydrazyl (DPPH) scavenging assay, which can be related to the high radical-scavenging activities of its phenolic contents. In addition, the AM antioxidant potential has been proved by measuring ABTS radical cation scavenging and DPPH scavenging (13, 14).

In line with the current findings, a study (45) using rats with alcohol-induced liver damage found that the plasma MDA concentration decreased and the activity of the antioxidant enzymes including GPx and SOD enhanced following AM extract treatment. Also, the use of 50, 100, and 200 mg/kg of AM extract was associated with an increased SOD activity and decreased MDA levels in mice (53). Recently, Asghari Baghkheirati et al. (54) showed that the use of 10 and 20 g/kg of AM powder in broilers diet reduces the MDA concentration in meat.

It is well-documented that a higher intake of natural antioxidants led to improved antioxidant status through transferring of these molecules to animal tissues (10), which leads to high-value food products (e.g., meat) and contributes to the meeting customer desires over safety and quality of products (49). Hence, improved GPx, SOD, and CAT along with mitigated MDA concentration in camels receiving AM-containing diets demonstrated that it could be a useful tool against oxidative damage.

## Conclusion

Overall, the results from this study showed that dietary inclusion of AM at levels of 25 or 50% DM of diet in growing camels increased DMI. The highest levels of dietary AM were associated with lower weight gain; however, the production costs were reduced by AM inclusion, and return per kg BW gain was improved. Plasma concentration of TG and CHOL was suppressed as well as hepatic enzyme including ALT, AST, and ALP, whereas antioxidant status elevated as indicated by higher TAC, GPx, SOD, and CAT. Under the conditions of this study, AM (up to 25% DM of diet) appears to have benefitted the immune system without deleterious effects on growing camel performance. For greater scientific reach, further studies should consider the use of AM focusing on rumen function and quality of animal products, such as meat.

## Data Availability Statement

The original contributions presented in the study are included in the article, further inquiries can be directed to NG, navid.ghavipanje@birjand.ac.ir.

## Ethics Statement

The animal study was reviewed and approved by Animal Welfare and Ethical Review Board of Camel and Range Species Research Station, Birjand, Iran.

## Author Contributions

NG and SH: conceptualization. NG, SH, and AA: methodology. NG, AV, and EV-B-P: validation and writing—review and editing. NG: formal analysis, visualization, project administration, and writing—original draft preparation. NG and MA: investigation. NG, AA, AV, and MA: data curation. All authors have read and agreed to the published version of the manuscript.

## Conflict of Interest

The authors declare that the research was conducted in the absence of any commercial or financial relationships that could be construed as a potential conflict of interest.

## Publisher's Note

All claims expressed in this article are solely those of the authors and do not necessarily represent those of their affiliated organizations, or those of the publisher, the editors and the reviewers. Any product that may be evaluated in this article, or claim that may be made by its manufacturer, is not guaranteed or endorsed by the publisher.
